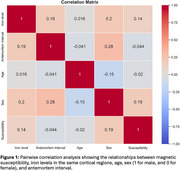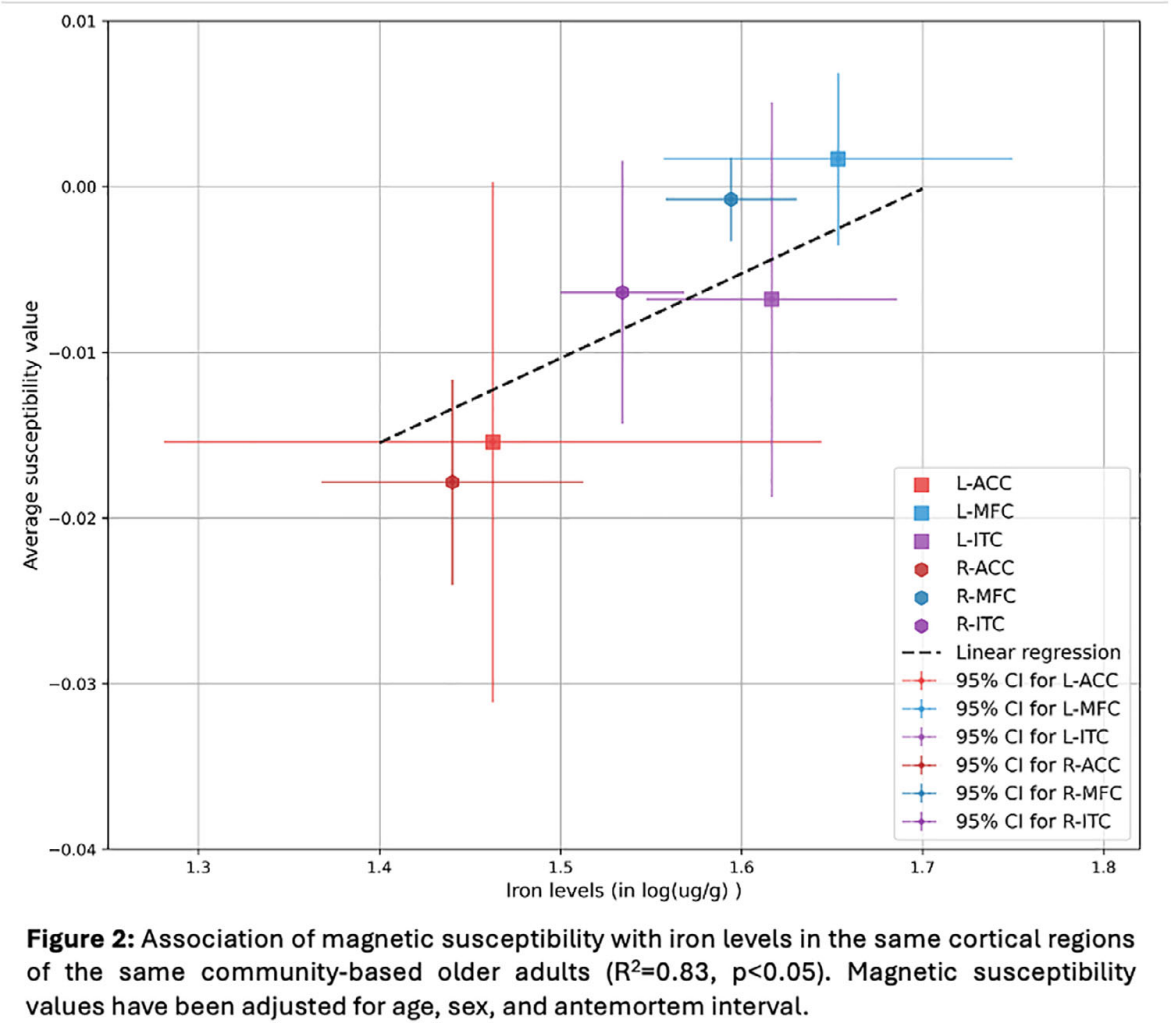# Association Between Cortical Magnetic Susceptibility and Iron Levels in Community‐Based Older Adults: A Quantitative study of in‐vivo and post‐mortem analysis

**DOI:** 10.1002/alz70856_097948

**Published:** 2025-12-24

**Authors:** Rasheed Abid, Md Tahmid Yasar, Abdur Raquib Ridwan, Mohammad Rakeen Niaz, Yingjuan Wu, Shengwei Zhang, Ashley I. Bush, Scott Ayton, Arnold M Evia, David A. A. Bennett, Julie A Schneider, Konstantinos Arfanakis

**Affiliations:** ^1^ Illinois Institute of Technology, Chicago, IL, USA; ^2^ Rush University Medical Center, Chicago, IL, USA; ^3^ Rush Alzheimer's Disease Center, Rush University Medical Center, Chicago, IL, USA; ^4^ The Florey Institute of Neuroscience and Mental Health, The University of Melbourne, Australia, Melbourne, VIC, Australia

## Abstract

**Background:**

Quantitative Susceptibility Mapping (QSM) is a critical tool for studying brain iron homeostasis and brain abnormalities. While some studies have analyzed autopsy‐derived iron concentrations alongside in‐vivo magnetic susceptibility in various brain regions, the relationship between post‐mortem iron levels and in‐vivo magnetic susceptibility remains insufficiently understood, especially in cortical regions. This study aims to examine the relationship between cortical magnetic susceptibility and iron levels in community‐based older adults.

**Methods:**

This study utilized 3D multi‐echo gradient echo (ME‐GRE) data from 20 community‐based older adults (ages 71–101 years, 16 female). Susceptibility maps were reconstructed using the Morphology Enabled Dipole Inversion (MEDI) algorithm. Multi‐atlas segmentation was performed on the T1‐weighted images and cortical regions were defined in the subject's native space, using the ANTS image registration toolbox. All participants underwent autopsy. The antemortem interval was 0.4 to 2.4 years (mean = 1.3 years). One cerebral hemisphere from each participant was studied (14 left, 6 right), and the contralateral hemisphere was frozen. Tissue samples from three cortical regions: anterior cingulate cortex (ACC), middle frontal cortex (MFC), and inferior temporal cortex (ITC) were collected. Iron concentrations were measured via inductively coupled plasma mass spectrometry and were log‐transformed to alleviate skewness. The median magnetic susceptibility for each region was recorded. Pairwise cross‐correlation assessed relationships between magnetic susceptibility, iron levels, age, sex, and antemortem interval. Multiple linear regression analysis examined the association between magnetic susceptibility and iron concentrations, adjusting for age, sex, and antemortem interval.

**Results:**

Pairwise correlation analysis revealed strong relationships between iron levels and magnetic susceptibility (Figure 1). Multiple linear regression showed a significant positive association between magnetic susceptibility and iron levels, controlling for age, sex, and antemortem interval, with R^2^ = 0.83, *p* < 0.05 (Figure 2).

**Conclusion:**

This study established a significant positive association between cortical magnetic susceptibility and iron levels in the cortex in the same community‐based older adults. The data from both in‐vivo QSM and post‐mortem tissue analysis suggest the potential for estimation of iron concentrations based on magnetic susceptibility values, with applications in future studies of iron dysregulation in neurodegenerative diseases.